# Ultra-high Performance Liquid Chromatography–Ion Mobility–High-Resolution Mass Spectrometry to Evaluate the Metabolomic Response of Durum Wheat to Sustainable Treatments

**DOI:** 10.1021/acs.jafc.3c04532

**Published:** 2023-10-05

**Authors:** Nicolò Riboni, Federica Bianchi, Monica Mattarozzi, Marina Caldara, Mariolina Gullì, Sara Graziano, Elena Maestri, Nelson Marmiroli, Maria Careri

**Affiliations:** † Department of Chemistry, Life Sciences and Environmental Sustainability, University of Parma, Parco Area delle Scienze 11/A-17/A, 43124 Parma, Italy; ‡ Center for Energy and Environment (CIDEA), Centro Santa Elisabetta, University of Parma, Parco Area delle Scienze 95, 43124 Parma, Italy; § Interdepartmental Center SITEIA.PARMA, University of Parma, Parco Area delle Scienze 181/A, 43124 Parma, Italy; ∥ Centro Santa Elisabetta, National Interuniversity Center for Environmental Sciences (CINSA), Parco Area delle Scienze 95, 43124 Parma, Italy

**Keywords:** ultra-high performance liquid chromatography–high-resolution mass spectrometry, ion mobility, untargeted metabolomics, multivariate data analysis, durum wheat, biostimulants, soil amendments

## Abstract

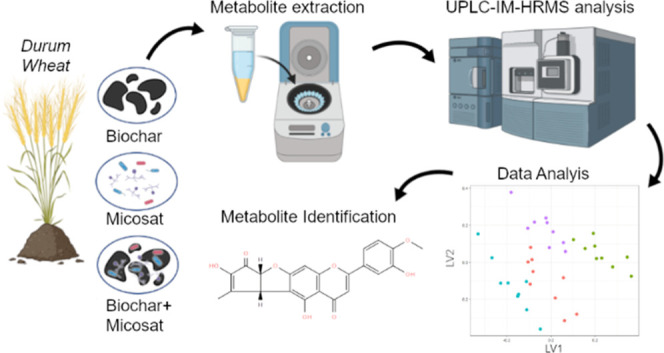

Sustainable agriculture aims at achieving a healthy food production while reducing the use of fertilizers and greenhouse gas emissions using biostimulants and soil amendments. Untargeted metabolomics by ultra-high performance liquid chromatography–ion mobility–high-resolution mass spectrometry, operating in a high-definition MS^E^ mode, was applied to investigate the metabolome of durum wheat in response to sustainable treatments, i.e., the addition of biochar, commercial plant growth promoting microbes, and their combination. Partial least squares-discriminant analysis provided a good discrimination among treatments with sensitivity, specificity, and a non-error rate close to 1. A total of 88 and 45 discriminant compounds having biological, nutritional, and technological implications were tentatively identified in samples grown in 2020 and 2021. The addition of biochar-biostimulants produced the highest up-regulation of lipids and flavonoids, with the glycolipid desaturation being the most impacted pathway, whereas carbohydrates were mostly down-regulated. The findings achieved suggest the safe use of the combined biochar-biostimulant treatment for sustainable wheat cultivation.

## 1 Introduction

Metabolomics is based on cutting-edge analytical techniques providing a snapshot of small molecules present in complex biological samples.^1^ Both targeted and untargeted metabolomics experiments can be carried out. In contrast to targeted metabolomics, which is focused on the identification and quantitation of a limited number of defined metabolites, the untargeted approach aims at acquiring data related to all ions within a certain mass range, thus providing a snapshot of most of the small molecules present in complex biological samples. Advances in high-resolution mass spectrometry (HRMS) permit a comprehensive profiling of food samples, representing a valuable technique for studying the metabolic changes developed by organisms in the presence of variable environmental factors. Ultra-high performance liquid chromatography coupled to HRMS (UHPLC-HRMS) currently represents the best tool to face challenges related to the complexity of metabolome.^2^ UHPLC provides efficient chromatographic separation of compounds, whereas HRMS is characterized by unparalleled specificity, sensitivity, and availability of large spectral databases. In addition, ion mobility spectrometry (IMS) offers great potential for improving depth of coverage in metabolomic studies, separating ions according to their collisional cross-section (CCS) and providing unique features for metabolite identification.^3^

Metabolomics has increasingly been applied to detect alterations in plants exposed to different treatments.^4,5^ Due to its nutritional relevance with 12–16% protein content, 70% carbohydrate, 1.9% fat, 1.6% fiber, 1.6% minerals, and other essential nutrients including antioxidants,^6^ together with the ease of handling, cooking, long shelf life, and high digestibility, durum wheat is suitable for pasta making and human consumption. Although it is a principal staple food for human nutrition and one of the most important and extensive crop species in the Mediterranean region, the studies involving the metabolomics of durum wheat are less than 10% of those related to wheat, which are mainly focused on bread wheat or other *Triticum* species.^5,7−9^

Recently, there has been growing awareness of the potential of metabolomics in environmental sciences and its management. In fact, environmental metabolomics is a rapidly maturing bioanalytical technique that can be applied for sustainable agriculture to understand crop response to stress by producing plant metabolites.^5^

Environmental sustainability aims to a healthy food production while reducing the use of fertilizers and greenhouse gas emissions.^10−12^ Improved soil health is recognized as a contribution to make agriculture more sustainable and the use of biostimulants and soil amendments participate in the development of the so-called climate smart agriculture.^13^ Among biostimulants, the plant growth promoting microbes (PGPM) can increase the bioavailability of nutrients in soil,^14^ while protecting crops from contaminants and pathogens.^15−18^ To improve the performance of biostimulants, it is possible to combine PGPM with soil amendments, which can affect soil fertility in terms of nutrient retention capacity and water filtration.^19^ Biochar, obtained by pyrolysis or pyro-gasification of renewable resources, is a good amendment especially when inoculated with PGPM, improving plant growth, yield, stress tolerance, adsorption of nutrients, and acting as a CO_2_ sink.^19−21^

An important issue concerns the effects of biostimulants and amendments on food quality. The role of PGPM in modifying crop quality in terms of nutritional content and technological properties by triggering the synthesis of beneficial metabolites has not yet been elucidated. In fact, up until now, most of the investigations have been focused on the role of PGPM in improving environmental stress response and crop yield.^22−25^

In this study, UHPLC-IMS-HRMS was applied for the first time to assess metabolomic response to different sustainable treatments in field trials: the addition of biochar, commercial PGPM (Micosat_F1) and a combination of biochar/Micosat_F1. Multivariate data analysis was applied to identify biomarkers able to differentiate among treatments, highlighting the triggered metabolic pathways, and supporting the decision-making process for a more sustainable agri-food environment.

## 2 Materials and Methods

### 2.1 Chemicals and Materials

LC-MS grade water, acetonitrile, methanol, and formic acid were purchased from Honeywell Burdick & Jackson (Charlotte, NC). Leucine enkephalin standard was from the Waters TOF G2-S Sample Kit-1 (Waters, Milford, MA).

### 2.2 Plant Material and Growth Conditions

Durum wheat (*Triticum durum* Desf., cultivar Svevo) was released by Produttori Sementi Bologna PSB S.p.A. (Italy) in 1996. The wheat field trials were performed at the experimental farm Stuard (Lat. 44_4802300N; Long. 10_1603000E; 58 m above sea level), close to the city of Parma (Italy). Meteorological parameters were collected daily by an automatic weather station installed in the neighboring experimental field during both growing seasons.

The Svevo cultivar was sown at a density of 400 seeds m^–2^ during the winter period. The experiment was set up in plots of 3 m^2^ (eight rows, 0.20 m between row distance, and 3 m long) always considering three replicated treatments *per* year. The conditions tested were: (i) CTR, control condition, having the soil treated with only 50 kg ha^–1^ of N (urea) instead of the usual fertilization (150 kg urea, 60 g ammonium nitrate, 60 kg phosphates) ha^–1^. All parcels were treated in the same way, with no supplement of organic fertilization (animal manure, pig slurry); (ii) CHAR, biochar obtained by slow pyrolysis of wood pellet,^20^ was applied before sowing at a rate of 0.25 kg m^–2^ (2.5 ton ha^–1^) and buried at a depth of 10 cm; (iii) Micosat_F1, granular microbial mix including both bacteria and fungi, provided by CCS Aosta (Aosta, Italy), was applied at the ratio 1:1 *w*/*w* per gr of seeds (which corresponds to 20 g m^–2^) (composition of Micosat_F1 is given in Table S1); (iv) CHAR_Micosat_F1, biochar with the addition of Micosat_F1 in the same amount described for the previous conditions. The experiments were performed during two harvesting years, namely, 2020 and 2021.

### 2.3 Metabolomic Analysis

#### 2.3.1 Metabolite Extraction

About 100 g of Svevo grains were randomly sampled from each condition, then the samples were freeze-dried, milled using Knifetec 1095 (Foss, Hillerød, Denmark) for 60 s at 4 °C, passed through a 0.5 mm sieve, and stored at −80 °C until analysis. Fifty mg of wheat flour and 2 mL of methanol 70% (*v*/*v*) were vortexed for 10 min at 2000 rpm, then the samples were extracted by ultrasonic assisted solvent extraction (Argo Lab DU-06, Carpi (MO), Italy) at a power of 4 au at 4 °C for 30 min. After extraction, the samples were centrifuged at 14,000*g* for 10 min at 4 °C, and the supernatant was collected, filtered through nylon filtering membrane 0.2 μm (Phenomenex, Torrance, CA) and submitted to UHPLC- IMS-HRMS analysis. For each year, a sample obtained by pooling the extracts of all the treatments was used as the quality control (QC) sample.

#### 2.3.2 Ultra-high Performance Liquid Chromatography–Ion Mobility–High-Resolution Mass Spectrometry

Untargeted metabolomics was performed using a binary Acquity UPLC I-Class system (Waters) coupled to a Waters Synapt G2-Si HDMS QTOF mass spectrometer equipped with an electrospray ionization (ESI) ZsprayTM (Waters) by operating both in positive and negative ion modes. Reversed phase chromatographic separation was carried out using a Kinetex 2.6 μm PS C18 100 Å (100 × 2.1 mm^2^) column (Phenomenex, Torrance, CA), maintained at 40 °C. The operating conditions were as follows: solvent A, water with 0.1% (*v*/*v*) formic acid; and solvent B, acetonitrile with 0.1% (*v*/*v*) formic acid. The flow rate was 0.4 mL min^–1^ and the injection volume was 2 μL. The multistep linear gradient elution started with solvent B set at 2% for 2 min, followed by a linear gradient to 50% within 9 min, then to 85% in 13 min, to 95% in 19.5 min maintained for 0.5 min before column re-equilibration (4 min). Electrospray conditions were as follows: capillary voltage, 0.80 and 0.50 kV in ESI^+^ and ESI^–^ respectively; cone voltage, 50 V; source temperature, 150 °C; source offset, 80 V; desolvation temperature, 600 °C; cone gas, 50 L h^–1^; desolvation gas, 800 L h^–1^; nebulizer pressure, 6.5 bar. IMS-HRMS analyses were performed at a mass resolution of 20000 fwhm (full width at half-maximum) and using a traveling wave (TWIM) as a drift cell. Ion mobility resolution was ∼45 (Ω/ΔΩ) as reported in the literature for TWIMS-based instruments.^26^ Nitrogen was used as the drift gas at a flow rate of 90 mL min^–1^, transfer wave velocity was set at 215 m s^–1^, wave velocity at 650 m s^–1^, and wave height at 40 V. The Major Mix IMS/Tof Calibration Kit (Waters) - mass range: 151.1–1966.9 Da; CCS: 130.4–372.6 Å^2^ was used for the CCS calibration in both positive and negative ion modes (Table S1). CCS calibration^27^ was automatically performed by the IntelliStart software using the MassLynx platform. A detailed description of the calibration procedure is reported in Table S1. The calibration settings are used throughout the analysis. A leucine enkephalin solution (50 ng mL^–1^ in acetonitrile/water, 50:50 (*v*/*v*) with 0.1% formic acid) was used as the lock mass. Spectra were acquired operating in the data-independent High-Definition MS^E^ acquisition mode using dynamic range enhancement and a collision energy ramp from 25 to 45 V for the high energy profile.

The UHPLC-IMS-HRMS data were recorded in raw files by using the MassLynx (v4.2) software (Waters). Data analysis was performed by processing the raw data using the Progenesis QI software (Waters, Milford, MA) as follows: auto- and manual alignment of signals, peak peaking, deconvolution, and normalization. The following adducts were considered: [M + H]^+^, [M + Na]^+^, [M + K]^+^, [M + NH_4_]^+^, [M + H_2_O + H]^+^, [M – H_2_O + H]^+^, [M + 2H]^2+^, [M + 2Na]^2+^, [M + 2Na–H]^+^, [2M + H]^+^, [2M + Na]^+^, [M + H + Na]^2+^ in ESI^+^, and [M – H]^−^, [M + H_2_O–H]^−^, [M – H_2_O–H]^−^, [M + HCOO]^−^, [2M – H]^−^, [M + Na-2H]^−^, [M + K-2H]^−^ in ESI^–^. The data were filtered by setting a maximum intragroup variability of 20%, a power analysis value >0.8, and a minimum fold change of 3 compared to a method blank.

Multivariate data analysis, namely, principal component analysis (PCA) and Partial least squares-discriminant analysis (PLS-DA), were performed on Pareto scaled data using open-source software Rstudio version 2022.12.0 Build 353 using the following packages: tidyverse,^28^ mdatools,^29^ caret,^30^ ggpubr,^31^ ggfortify,^32^ MetabolAnalyze,^33^ devtools,^34^ and rstatix.^35^ PCA was performed to explore the data set and to obtain the features able to differentiate samples belonging to the different agro-treatments. PLS-DA followed by *k*-fold cross-validation (*k* = 5, repeat *n* = 50) was performed. Variable importance in projection (VIP analysis) was used to rank metabolites according to their discrimination potential (VIP score ≥2) with the final aim of reducing the number of discriminating compounds. A fold change >1.2 and <0.8 was used for the assessment of up- and down-regulation, respectively.

Compound identification was performed by comparing the spectra with those stored in online libraries, namely, Human Metabolome Database, Food Metabolome Database, PlantCyc, Carotenoid Database, LIPID MAPS, Phenol Explorer Database, and KEGG Database, using a mass error tolerance of 5 ppm for precursor ions and 10 ppm for fragment ions. An isotope similarity threshold of 80% was set. To increase the annotation reliability, the CCS values obtained in this study were compared, using a 5% threshold value, with those stored in homemade databases or predicted by machine learning algorithms, also present in HMDB among which are AllCCS^36^ and DeepCCS.^37^ When possible, a comparison of experimental CCS values was performed with those obtained by the analysis of standards. Discriminant compounds were analyzed in PlantCyc^38^ to highlight enriched metabolic pathways. The LIPID MAPS glycerophospholipid abbreviations (PC, PE, etc.) are used here to refer to the annotated analytes.

Finally, compound heatmaps were computed using MATLAB release R2023a.^39^

## 3 Results and Discussion

In this study, a metabolomic analysis on grains collected from durum wheat (cv Svevo) grown by applying different sustainable treatments was carried out to provide more insights into the effects of biostimulants and biochar addition both on the pattern and on the amount of metabolites in durum wheat flours. Being commercially available, with a worldwide gross market, both Micosat_F1 and biochar were selected as biostimulant and amendment, respectively. Most applications in the last years were on potato, tomato, and other horticultural crops; however, there was a growing interest in testing their effects also on commodity crops like wheat, performing field trials, like in our case. To study the metabolome, the use of IMS combined with HRMS provided significant improvements compared to the use of the sole HRMS since it allowed the determination of the drift for each detected feature, allowing the calculation of CCS values, which is a compound-specific parameter that could be used as an additional identification parameter for feature annotation and compound identification.^40^ In fact, this parameter can decrease the number of false positive annotations and resolve ambiguity among isomers and isobaric species, especially by considering the matching of the CCS of the different adduct species for the same feature (if present).^41^ In addition, a third separation dimension is added to the system, thus allowing a more effective separation of metabolites, especially for such a complex biological mixture. This additional separation decreases the background noise, and it is not affected by the matrix effect,^41^ thus improving the reliability of the analysis.

### 3.1 Multivariate Data Analysis

UHPLC-IMS-HRMS analyses were performed to identify metabolites able to differentiate among the applied treatments. The use of the MS^E^ acquisition mode, based on alternating scanning acquisitions at both low and high energies, provided useful information on both precursor and fragment ions within a single run.^42^ Additionally, the capability of IMS was exploited to add an extra dimension to the separation of complex samples, also increasing the confidence in analyte identification via calculation of the CCS values.

To reduce the number of features produced by IMS-HRMS and identify only those able to differentiate among the treatments, filtering and data reduction strategies in terms of intra-group variability, minimum fold change, and statistical power were applied, thus obtaining a total of 3587 and 4686 features for Svevo 2020 and Svevo 2021, respectively.

**Figure 1:**
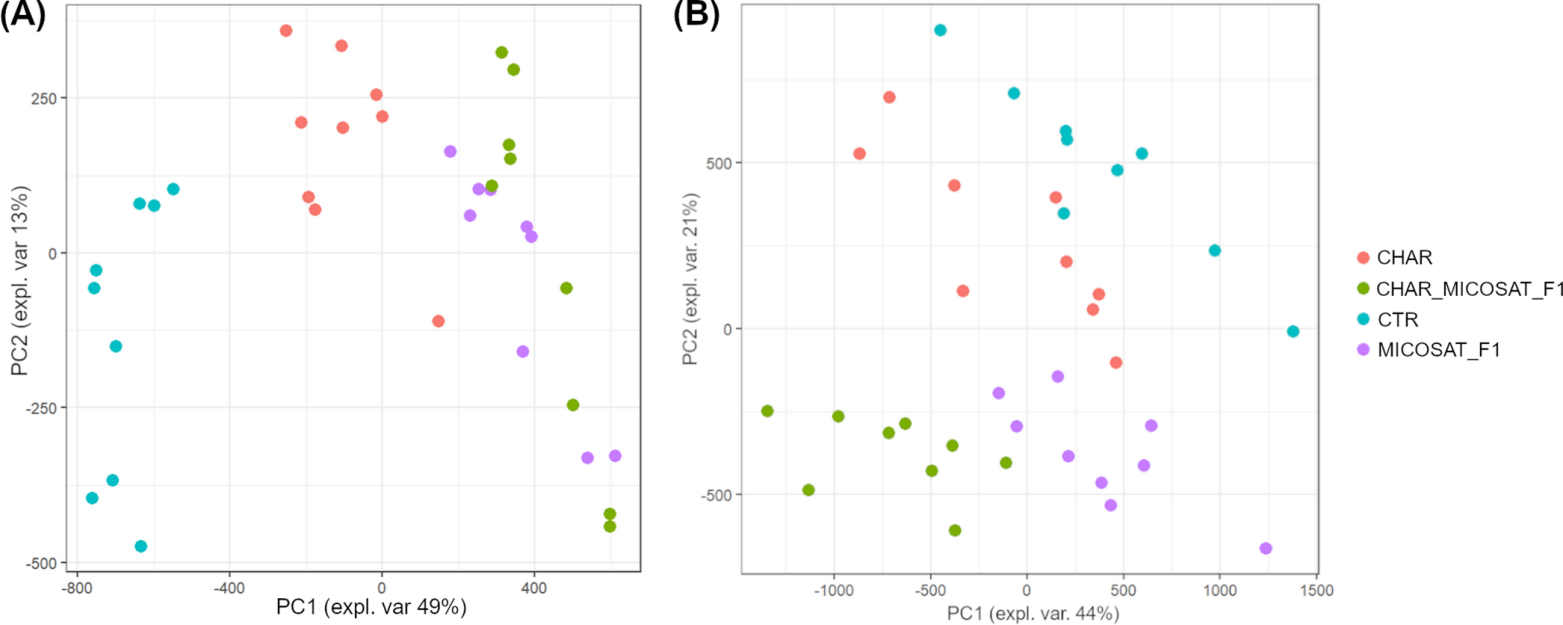
Principal component analysis of metabolomic data related to the wheat flour of (A) Svevo 2020 and (B) Svevo 2021 cultivated by applying different treatments.

First, an explorative PCA was carried out to detect possible clusters within samples: PC1 and PC2 explained 62 and 65% of the total variance for Svevo 2020 and Svevo 2021, respectively (Figure 1).

**Figure 2:**
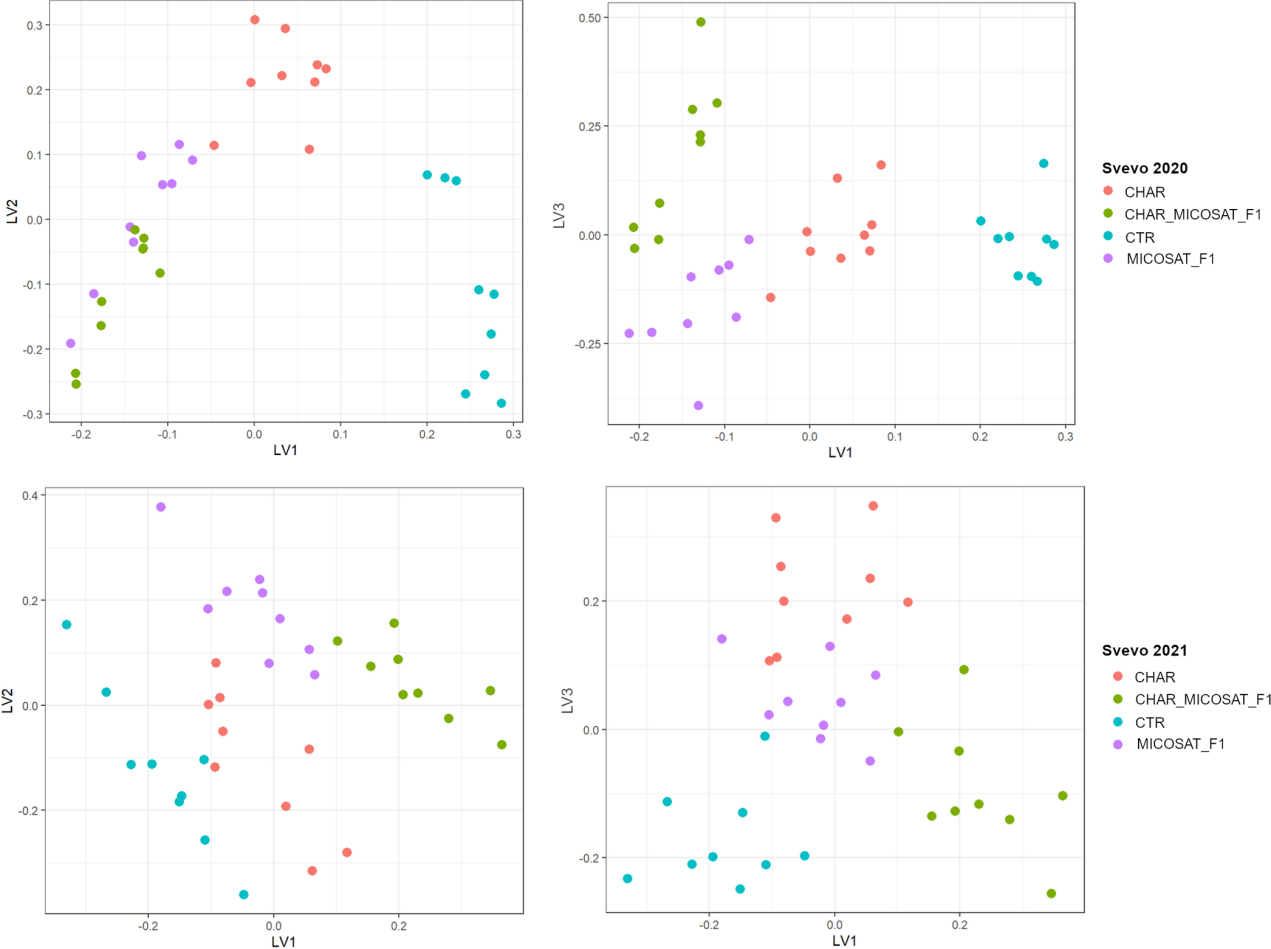
PLS-DA on the metabolomic data of Svevo 2020 (top) and Svevo 2021 (bottom).

Since only a partial differentiation among the treatments for both harvesting years was observed, a supervised PLS-DA pattern recognition approach was applied. Selecting six latent variables, a good discrimination among the treatments was feasible, as shown in Figure 2.

Table 1 summarizes the classification parameters, namely, sensitivity (Sn), specificity (Sp), non-error rate (NER), representing the ability to correctly classify samples in their respective classes, the ability to reject samples belonging to other classes, and the average of class sensitivities, respectively.^43^ All calculated parameters were close to 1, proving the good performance of the model in both the fitting and cross-validation.

**Table 1:** Sensitivity, specificity, and non-error rate of the models in fitting and cross-validation for Svevo 2020 and Svevo 2021 samples

		CTR		Micosat_F1		CHAR		CHAR_Micosat_F1	
	NER^a^	Sn^b^	Sp^c^	Sn^b^	Sp^c^	Sn^b^	Sp^c^	Sn^b^	Sp^c^
Svevo 2020									
fitting	1	1	1	1	1	1	1	1	1
cross-validation	0.94	1	1	0.89	1	0.89	0.96	1	1
Svevo 2021									
fitting	1	1	1	1	1	1	1	1	1
cross-validation	0.96	0.89	1	0.96	0.56	1	0.96	1	1

a NER: non-error rate.

b Sn: sensitivity.

c Sp. Specificity.

VIP analysis was applied to reduce the number of discriminatory metabolites to be identified, according to VIP score criteria >2, 265, and 285 metabolites for the Svevo 2020 and Svevo 2021 samples were obtained, respectively. The corresponding features were submitted to identification considering the information derived from accurate mass measurements of both parent and fragment ions, fragmentation and isotopic patterns, library matching, score fit, and CCS values. As for CCS values, the match between the experimental values calculated in this study with those contained in public databases or predicted by machine learning algorithms was used to obtain the proper level of identification (level 2). When the injection of standards was feasible, the obtained values allowed for a level 1 identification.

### 3.2 Metabolomic Profiling

**Figure 3:**
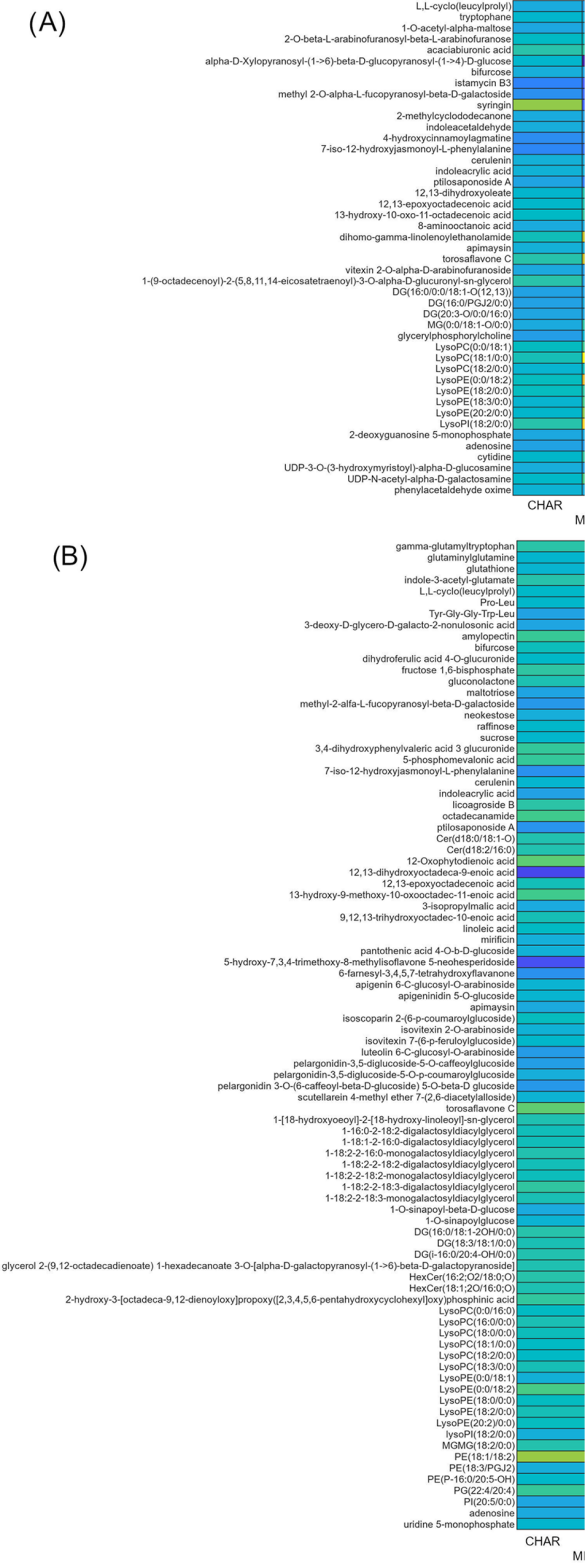
Heat map representing the regulation of the annotated metabolites in (A) Svevo 2020 and (B) Svevo 2021. The rows display metabolites, and the columns represent the different treatments. Color-coded scale from blue to yellow indicates the variation in the range from 0.2 to +2 of down- and up-accumulated metabolites compared to the control.

A total of 45 and 88 metabolites belonging to different classes were tentatively identified in Svevo grains cultivated in 2020 and 2021, respectively (Table S1). Glycerophospholipids, carbohydrates, and carbohydrate conjugates were the most abundant chemical classes in Svevo 2020, accounting for 18 compounds, whereas glycerophospholipids, glycerolipids, flavones and flavonoids, carbohydrates and carbohydrate conjugates, fatty acyls, carboxylic acids, and derivatives were the most abundant chemical classes in Svevo grain cultivated in 2021, accounting for 77 compounds. The annotated VIP compounds were submitted to fold change analysis to describe direction and intensity of regulation. In fact, most of the metabolites play a key role in the metabolomic processes; as shown in Figure 3, the use of different treatments was able to affect the metabolite regulation only in a very slight manner compared with the control conditions. As for Svevo 2021 samples, the applied treatments produced modest up- and down-regulations of most of the compounds (Figure 3B). Considering a fold change >1.2, 36, 11, and 20% of the annotated features for CHAR_Micosat_F1, CHAR, and Micosat_F1, respectively, were up-regulated, whereas 17, 9, and 13% of down-regulation was observed. Among the investigated treatments, CHAR_Micosat_F1 produced a general increase in up-regulation for almost all the chemical classes considered, with the sole exception of carbohydrates and carbohydrate conjugates. By contrast, when Svevo 2020 samples were considered, the percentage of up-regulated compounds was 31, 7, and 24% for CHAR_Micosat_F1, CHAR and Micosat_F1, respectively, vs 22, 11, and 24% of down-regulations. As represented in Figure 3A, also in this case, small changes in regulation were observed. A significant difference in terms of number of annotated compounds was observed between the two harvesting years. Since environmental and meteorological factors can affect the biosynthesis of various plant metabolites, these findings could be related to the different weather conditions of the growing seasons. As regard to this aspect, in 2020, a total rainfall of 186 mm was observed, with temperature ranging between −3.5 and +33.5 °C, whereas in 2021, the total rainfall was 150 mm, with temperatures recorded in the −6.9 to + 36.2 °C range. It is known that water shortage and persistence of heat during grain filling can constitute stressful conditions, which affect both plant development and wheat quality, depending on both the timing and duration of the weather events.^44−46^ As reported in previous studies, drought conditions induced an increase in gluten strength, whereas heat stress exerted an opposite effect.^47,48^ Our findings demonstrated a slight up-regulation for 14 out of 46 lipids (30%) when the combined treatment CHAR_Micosat_F1 was applied to Svevo 2021 samples. A similar effect was observed for Svevo 2020 as 53% (10 compounds out of 19) of the lipid compounds were over-expressed when the CHAR_Micosat_F1 treatment was applied.

When the CHAR_Micosat_F1 treatment was used, more up-regulated lipids (53 and 30% for Svevo 2020 and Svevo 2021, respectively) as compared to the Micosat_F1 treatment (37 and 9%) were observed. Due its highly porous structure, CHAR provides a favorable habitat for microorganisms permitting their growth in the soil environment,^19^ being also a source of labile organic C which improves microorganisms metabolic activity, both bacteria and fungi. The bacterial component of Micosat_F1 which can benefit from this release is represented by strains that can promote plant growth improving phosphorus solubilization and nitrogen fixation and can mitigate biotic and abiotic stress (Table S1).

**Figure 4:**
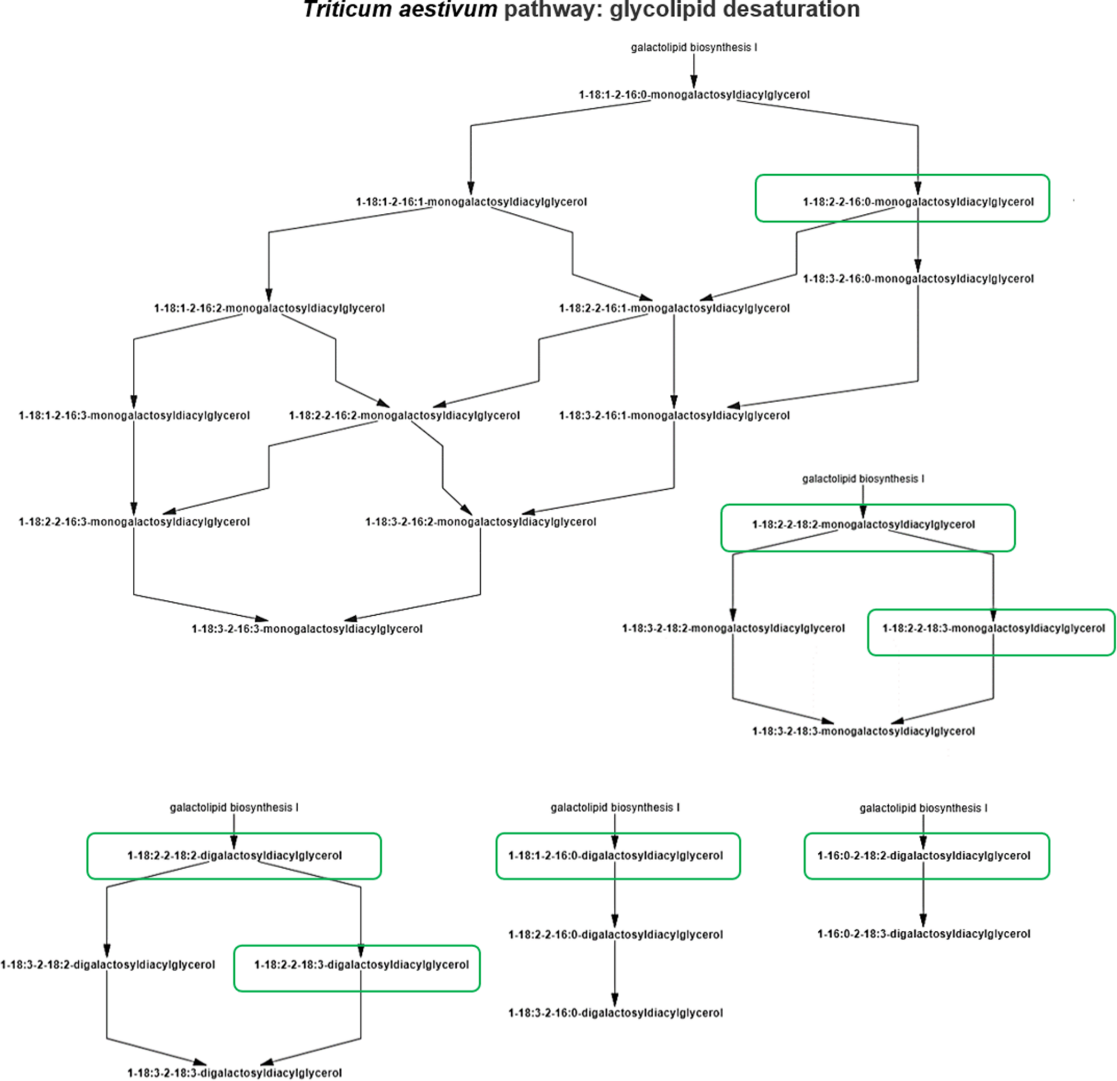
Pathway analysis: metabolites with a significant increase or decrease are evidenced (in green).

Moradi and co-workers^46^ showed that mycorrhization can enhance the expression of genes related to the lipid metabolism to maintain the integrity of cellular membranes, demonstrating that under stress conditions, plants are able to promote lipid biosynthesis, thus increasing the cell membrane thickness as a defense mechanism. Conversely, Bernardo et al.^49^ observed a down-accumulation of lipids in mycorrhizal roots under water stress conditions, ascribing this behavior to the consumption of lipids as the primary source of C by mycorrhizal fungi. Therefore, the most impacted pathway was glycolipid desaturation (Figure 4). As observed by matching the data set against the PlantCyc *Triticum aestivum* database, the composition of glycolipids and phospholipids accounts for most of the polar lipids of wheat flour, during dough development, glycolipids accounting for 93% of the polar lipids, being preferentially associated with glutenins via hydrogen bonds and hydrophobic interactions.^50^ Specifically, the glycolipid desaturation pathway synthesizes the α-linolenic acid via sequential steps of glycolipid-linked desaturation, being part of the synthesis of the hormone jasmonic acid. The function of jasmonic acid within a plant is very complex as it is involved in creating a balance between growth, development, and defense mechanisms of the plant.^51^

It should be noted that lipids can also affect the breadmaking quality of flour, thus playing a valuable role from a technological point of view. As demonstrated by Min and co-workers,^52^ the increase in the content of galactolipids, namely, monogalactosyl diglycerol and digalactosyl diglycerol in the flour and dough liquor was able to promote the stabilization of the gas cells formed during mixing and fermentation, leading to increased loaf volume and evenness of texture. Additionally, endogenous lipids in vital wheat gluten were found to affect the affinity of gluten proteins to water and the thermal characteristics of the dough, thus, influencing the quality of baked products. In fact, a higher water affinity and a lower denaturation temperature can lead to high-quality bakery products.^53^ The up-regulation of glycolipids and glycerophospholipids (57 and 37% for Svevo 2020 and Svevo 2021 samples, respectively) obtained by using the combined CHAR_Micosat_F1 treatment could be considered as a first step to produce higher quality flour for the production of bakery products. As already observed by Bernardo et al.,^49^ carbohydrates were generally down-regulated accounting for 45 and 50% when CHAR_Micosat_F1 was applied, whereas the use of Micosat_F1 resulted in a decrease of 27 and 38% for 2020 and 2021, respectively. In particular, α-d-Xylopyranosyl-(1-6)-β-d-glucopyranosyl-(1-4)-d-glucose, istamycin B3, and methyl-2-*O*-α-l-fucopyranosyl-β-d-galactoside were the down-regulated carbohydrates when both Micosat_F1 and CHAR_Micosat_F1 treatments were applied in Svevo 2020 samples. Similarly, amylopectin, methyl-2-alfa-l-fucopyranosyl-β-d-galactoside, and neokestose were expressed at low levels in Svevo 2021 samples. This could be ascribed to the use of these carbohydrates as an additional supply of C to mycorrhizal fungi. Similar findings were also observed by Wang and co-workers,^54^ who evaluated the regulation of nutrient exchange between plant hosts and arbuscular mycorrhizal symbiosis.

**Figure 5:**
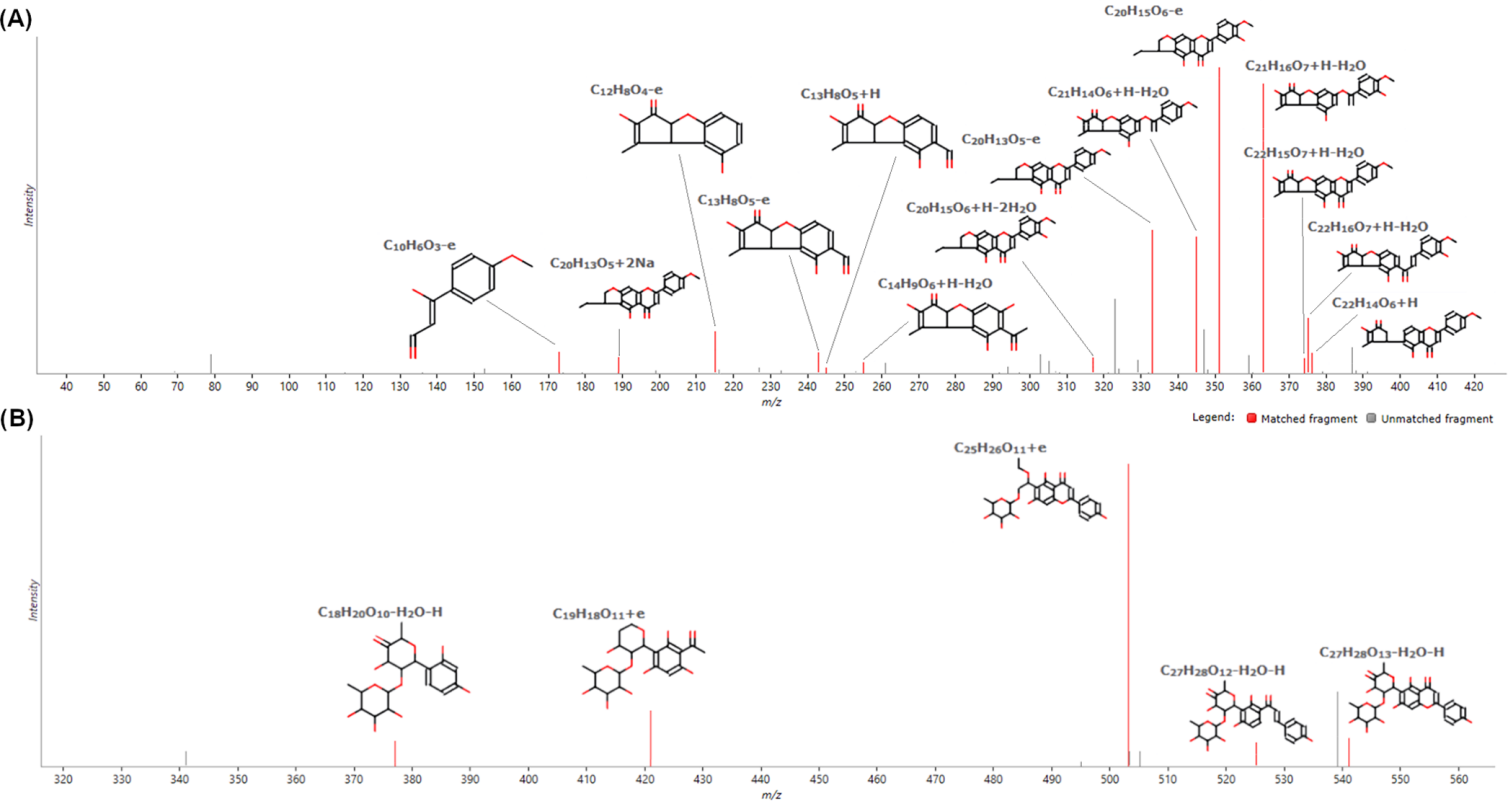
High-energy HRMS spectra of (A) torosaflavone C and (B) apimaysin. The structures of matched fragments are reported.

From the nutritional point of view, carbohydrates play a pivotal role in human diet by providing a high energy intake.^55^ Dietary fiber is important for human health, since it has impact on the utilization of grain and on the end-use quality, being used for the production of different functional foods.^56^ It is known that small fermentable carbohydrates, among which are raffinose and neokestose, are poorly absorbed in the small intestine, thus arriving at the colonic lumen, where they can exert a prebiotic effect but also trigger gastrointestinal symptoms in susceptible individuals. A diet characterized by a low intake of these metabolites can reduce fermentation in the colon, being recommended for people affected by irritable bowel syndrome and inflammatory bowel diseases.^57^ Finally, as shown in Figure 3, another interesting effect of biostimulation was exerted on flavonoids. Flavones and flavonoids are secondary metabolites having recognized antioxidant properties, which are proved beneficial in the treatment of different diseases.^55,58^ The number of compounds annotated as VIPs in the two harvesting years was very different, with only 3 compounds, namely, apimaysin, torosaflavone C, and vitexin 2-*O*-α-d-arabinofuranoside belonging to Svevo 2020 samples as compared to the 14 compounds annotated in Svevo 2021 samples. Apimaysin and torosoflavone C (Figure 5) were the only compounds common to both years.

Similar to lipids, a general up-regulation of flavones and flavonoids was observed in Svevo 2021 samples when Micosat_F1 and CHAR_Micosat_F1 were applied, accounting for 50 and 71%, respectively. The only exceptions were 5-hydroxy-7,3,4-trimethoxy-8-methylisoflavone-5-neohesperidoside and 6-farnesyl-3,4,5,7-tetrahydroxyflavanone, which resulted in down-regulation for all the treatments. Finally, CHAR addition produced a global down-regulation (29%) of flavones and flavonoids, with the exception of torosoflavone C which was up-regulated. As for Svevo 2020 samples, the up-regulation for torosoflavone C was still observed for both CHAR_Micosat_F1 and Micosat_F1, whereas vitexin 2-*O*-α-d-arabinofuranoside was slightly down-regulated. Previous studies demonstrated an increase in the flavonoid content during stress conditions to protect plants from a wide range of biotic and abiotic stresses, such as viruses, fungi, bacteria, ultraviolet radiation, salinity, or water loss;^5,59,60^ however, they can also act as chemical messengers in association with mycorrhiza.^58^ These findings could explain the general up-regulation of these compounds when Micosat_F1 was applied. This effect was enhanced for the combined CHAR_Micosat_F1 combined treatment. Both the reduced water availability and the high temperatures observed in 2021 could explain the highest number of flavones and flavonoids discriminating between the treatments in Svevo 2021 samples. In particular, it is known that the protective role of flavonoids can be related to their chemical structure, characterized by the presence of hydroxyl groups, double carbon bonds and modifications like glycosylation, prenylation and methylation.^61^ The effect of glycosylation is both to increase water solubility and to reduce flavonoids reactivity, thus being regarded as a valuable protection tool against cytoplasmic damage.^62^ Therefore, the presence of several glycosylated flavonoids in Svevo 2021 samples can be ascribed to the highest stress conditions in terms of both water deficiency and heat stress reached during this harvesting year.

As a general remark, it can be stated that the identification of specific pattern of metabolites for each treatment can be of paramount importance in assessing the quality of durum wheat, including its beneficial effects on health, thus allowing the agri-food systems to move toward green and climate resilient practices. Preliminary investigations (data not shown) in the rhizospheric microbial population did not reveal any significant reduction in biodiversity when CHAR_Micosat_F1 was used, thus ensuring its safe application in sustainable wheat cultivation. Further analyses dealing with soil variations, plant growth, and metagenomic analyses of roots are in progress to obtain deeper insights into the plant response after interactions with microbes. Additional expected benefits based on increased use of natural amendments and biofertilizers will rely on (i) the reduction in both water consumption and greenhouse gas emissions, (ii) a considerable decrease in the carbon footprint of food production, and (iii) a reduction of soil pollution due to a lower use of fertilizers. In this context, the achieved results could pave the way for assessing harmonized conditions based on the use of biofertilizers combined with biochar deriving from agricultural and/or food processing wastes to close the gap between the production and their consumption.

## Supporting Information Available

Information regarding Micosat_F1 composition, the analytes annotated in both Svevo 2020 and Svevo 2021 samples (Table S1) (XLSX)

## Abbreviations

CCS: collisional cross-section
CHAR: biochar
CHAR_Micosat_F1: biochar with the addition of Micosat_F1
CTR: control condition
ESI: electrospray ionization
HRMS: high-resolution mass spectrometry
IMS: ion mobility spectrometry
NER: non-error rate
PCA: principal component analysis
PGPM: plant growth promoting microbes
PLS-DA: partial-least-squares discriminant analysis
Sn: sensitivity
Sp: specificity
UHPLC: ultra-high performance liquid chromatography
VIP: variable importance in projection
